# Spontaneous Reaction Silencing in Metabolic Optimization

**DOI:** 10.1371/journal.pcbi.1000236

**Published:** 2008-12-05

**Authors:** Takashi Nishikawa, Natali Gulbahce, Adilson E. Motter

**Affiliations:** 1 Division of Mathematics and Computer Science, Clarkson University, Potsdam, New York, United States of America; 2 Department of Physics and Astronomy and Northwestern Institute on Complex Systems, Northwestern University, Evanston, Illinois, United States of America; 3 Department of Physics and Center for Complex Network Research, Northeastern University, Boston, Massachusetts, United States of America; 4 Center for Cancer Systems Biology, Dana Farber Cancer Institute, Boston, Massachusetts, United States of America; University of Washington, United States of America

## Abstract

Metabolic reactions of single-cell organisms are routinely observed to become dispensable or even incapable of carrying activity under certain circumstances. Yet, the mechanisms as well as the range of conditions and phenotypes associated with this behavior remain very poorly understood. Here we predict computationally and analytically that any organism evolving to maximize growth rate, ATP production, or any other linear function of metabolic fluxes tends to significantly reduce the number of active metabolic reactions compared to typical nonoptimal states. The reduced number appears to be constant across the microbial species studied and just slightly larger than the minimum number required for the organism to grow at all. We show that this *massive spontaneous reaction silencing* is triggered by the irreversibility of a large fraction of the metabolic reactions and propagates through the network as a cascade of inactivity. Our results help explain existing experimental data on intracellular flux measurements and the usage of latent pathways, shedding new light on microbial evolution, robustness, and versatility for the execution of specific biochemical tasks. In particular, the identification of optimal reaction activity provides rigorous ground for an intriguing knockout-based method recently proposed for the synthetic recovery of metabolic function.

## Author Summary

Cellular growth and other integrated metabolic functions are manifestations of the coordinated interconversion of a large number of chemical compounds. But what is the relation between such whole-cell behaviors and the activity pattern of the individual biochemical reactions? In this study, we have used flux balance-based methods and reconstructed networks of *Helicobacter pylori*, *Staphylococcus aureus*, *Escherichia coli*, and *Saccharomyces cerevisiae* to show that a cell seeking to optimize a metabolic objective, such as growth, has a tendency to spontaneously inactivate a significant number of its metabolic reactions, while all such reactions are recruited for use in typical suboptimal states. The mechanisms governing this behavior not only provide insights into why numerous genes can often be disabled without affecting optimal growth but also lay a foundation for the recently proposed synthetic rescue of metabolic function in which the performance of suboptimally operating cells can be enhanced by disabling specific metabolic reactions. Our findings also offer explanation for another experimentally observed behavior, in which some inactive reactions are temporarily activated following a genetic or environmental perturbation. The latter is of utmost importance given that nonoptimal and transient metabolic behaviors are arguably common in natural environments.

## Introduction

A fundamental problem in systems biology is to understand how living cells adjust the usage pattern of their components to respond and adapt to specific genetic, epigenetic, and environmental conditions. In complex metabolic networks of single-cell organisms, there is mounting evidence in the experimental [1]–[6] and modeling [7]–[14] literature that a surprisingly small part of the network can carry all metabolic functions required for growth in a given environment, whereas the remaining part is potentially necessary only under alternative conditions [15]. The mechanisms governing this behavior are clearly important for understanding systemic properties of cellular metabolism, such as mutational robustness, but have not received full attention. This is partly because current modeling approaches are mainly focused on predicting whole-cell phenotypic characteristics without resolving the underlying biochemical activity. These approaches are typically based on optimization principles, and hence, by their nature, do not capture processes involving non-optimal states, such as the temporary activation of latent pathways *during* adaptive evolution towards an optimal state [16],[17].

**Figure 1:**
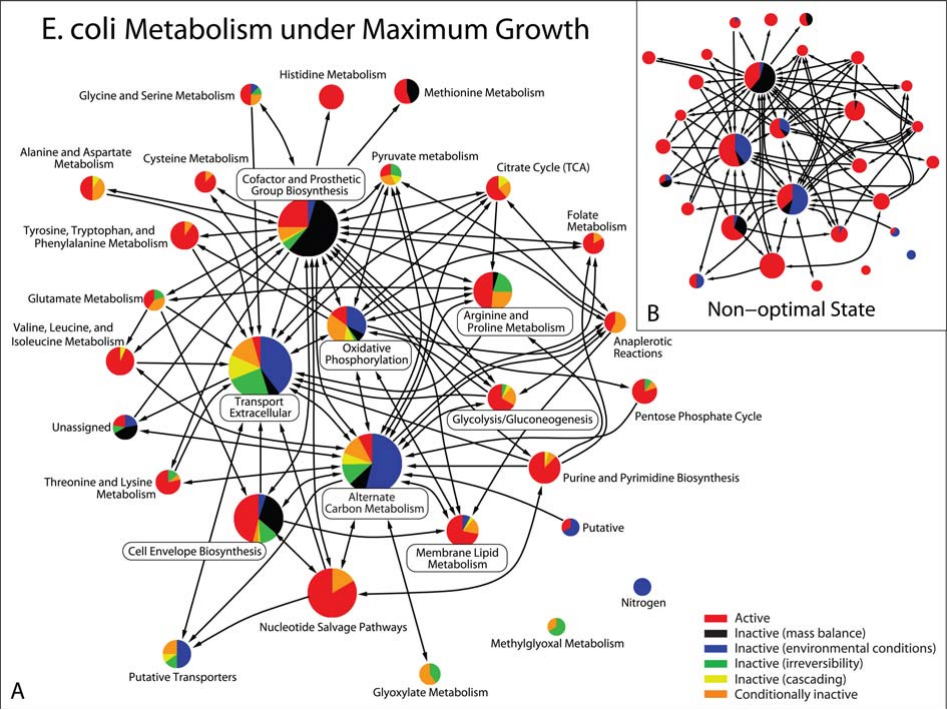
Optimal (A) and non-optimal (B) reaction activity in the reconstructed metabolic network of *E. coli* in glucose minimal medium (Materials and Methods). The pie charts show the fractions of active and inactive reactions in the metabolic subsystems defined in the iJR904 database [75]. The color code is as follows: active reactions (red), inactive reactions due to mass balance (black) and environmental constraints (blue), inactive reactions due to the irreversibility (green) and cascading (yellow) mechanisms, and conditionally inactive reactions (orange), which are inactive reactions that can be active for other growth-maximizing states under the same medium condition. The optimal state shown in panel A was obtained by flux balance analysis (FBA, see Materials and Methods). The network is constructed by drawing an arrow from one subsystem to another when there are at least 4 metabolites that can be produced by reactions in the first subsystem and consumed by reactions in the second. Larger pies represent subsystems with more reactions.

To provide mechanistic insight into such behaviors, here we study the metabolic system of single-cell organisms under optimal *and* non-optimal conditions in terms of the number of *active* reactions (those that are actually used). We implement our study within a flux balance-based framework [18]–[23]. Figure 1 illustrates key aspects of our analysis using the example of *Escherichia coli*. For any typical non-optimal state (Figure 1B), all the reactions in the metabolic network are active, except for those that are necessarily inactive due either to mass balance constraints or environmental conditions (e.g., nutrient limitation). In contrast, a large number of additional reactions are predicted to become inactive for any metabolic flux distribution maximizing the growth rate (Figure 1A). This spontaneous reaction silencing effect, in which optimization causes massive reaction inactivation, is observed in all four organisms analyzed in this study, *H. pylori*, *S. aureus*, *E. coli*, and *S. cerevisiae*, which have genomes and metabolic networks of increasing size and complexity (Materials and Methods). Our analysis reveals two mechanisms responsible for this effect: (1) *irreversibility* of a large number of reactions, which under intracellular physiological conditions [14] is shared by more than 62% of all metabolic reactions in the organisms we analyze (Table 1 and Note 1); and (2) *cascade of inactivity* triggered by the irreversibility, which propagates through the metabolic network due to stoichiometric and flux balance constraints. We provide experimental evidence of this phenomenon and explore applications to data interpretation by analyzing intracellular flux and gene activity data available in the literature.

**Table 1:** Reversibility of metabolic reactions in the reconstructed networks.

	*H. pylori*	*S. aureus*	*E. coli*	*S. cerevisiae*
Total number of reactions [*n*]	479	641	931	1149
Reversible	165	220	245	430
Irreversible	314	421	686	719

The drastic difference between optimal and non-optimal behavior is a general phenomenon that we predict not only for the maximization of growth, but also for the optimization of any typical objective function that is linear in metabolic fluxes, such as the production rate of a metabolic compound. Interestingly, we find that the resulting number of active reactions in optimal states is fairly constant across the four organisms analyzed, despite the significant differences in their biochemistry and in the number of available reactions. In glucose media, this number is ∼300 and approaches the minimum required for growth, indicating that optimization tends to drive the metabolism surprisingly close to the onset of cellular growth. This reduced number of active reactions is approximately the same for any typical objective function under the same growth conditions.

We suggest that these findings will have implications for the targeted improvement of cellular properties [24]. Recent work predicts that the knockout of specific enzyme-coding genes can enhance metabolic performance and even rescue otherwise nonviable strains [25]. The possibility of such knockouts bears on the issue of whether the inactivation of the corresponding enzyme-catalyzed reactions would bring the whole-cell metabolic state close to the target objective. Thus, our identification of a cascading mechanism for inducing optimal reaction activity for arbitrary objective functions provides a natural set of candidate genetic interventions for the knockout-based enhancement of metabolic function [25].

## Results

### Typical Nonoptimal States

We model cellular metabolism as a network of metabolites connected through reaction and transport fluxes. The state of the system is represented by the vector **v** = (*v*_1_,…,*v_N_*)*^T^* of these fluxes, including the fluxes of *n* internal and transport reactions, as well as *n*_ex_ exchange fluxes for modeling media conditions. Under the constraints imposed by stoichiometry, reaction irreversibility, substrate availability, and the assumption of steady-state conditions, the state of the system is restricted to a *feasible solution space* 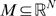 (Materials and Methods). Within this framework, we first consider the number of active reactions in a typical non-optimal state **v**∈*M*.

We can prove that, with the exception of the reactions that are inactive for all **v**∈*M*, all the metabolic reactions are active for almost all **v**∈*M*, i.e., for any typical state chosen randomly from *M* (Text S1, Section 1). Accordingly, the number *n*_+_(**v**) of active reactions in a typical non-optimal state is constant, i.e.,
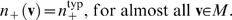
(1)The reactions that are inactive for all states are so either due to mass balance or environmental conditions, and can be identified numerically using flux coupling [26] and flux variability analysis [9].

#### Mass balance

Part of the metabolic reactions are forced to be inactive solely due to mass balance, independently of the medium conditions. For example, glutathione oxidoreductase in the *E. coli* reconstructed model involves oxidized glutathione, but because there is no other metabolic reaction that can balance the flux of this metabolite, the reaction cannot be active in any steady state. We characterize such reactions uniquely by a linear relationship between vectors of stoichiometric coefficients (Text S1, Section 2). Although these reactions are inactive in any steady state, some of them may play a role in transient dynamics (e.g., after environmental changes) [28], for which time-dependent analysis is required [29]. Others may be part of an incomplete pathway at an intermediate stage of the organism's evolution or, more likely, an artifact of the incompleteness or stoichiometric inconsistencies of the reconstructed model. Such inconsistencies have been identified in previous models [27], such as an earlier version of the model we use for *S. cerevisiae* [30].

#### Environmental conditions

Other reactions are constrained to be inactive due to the constraints arising from the environmental conditions imposed by the medium. For example, all reactions in the allantoin degradation pathway must be inactive for *E. coli* in media with no allantoin available, since allantoin cannot be produced internally. Similarly, the reactions involved in aerobic respiration are generally inactive for any state under anaerobic growth.

**Figure 2:**
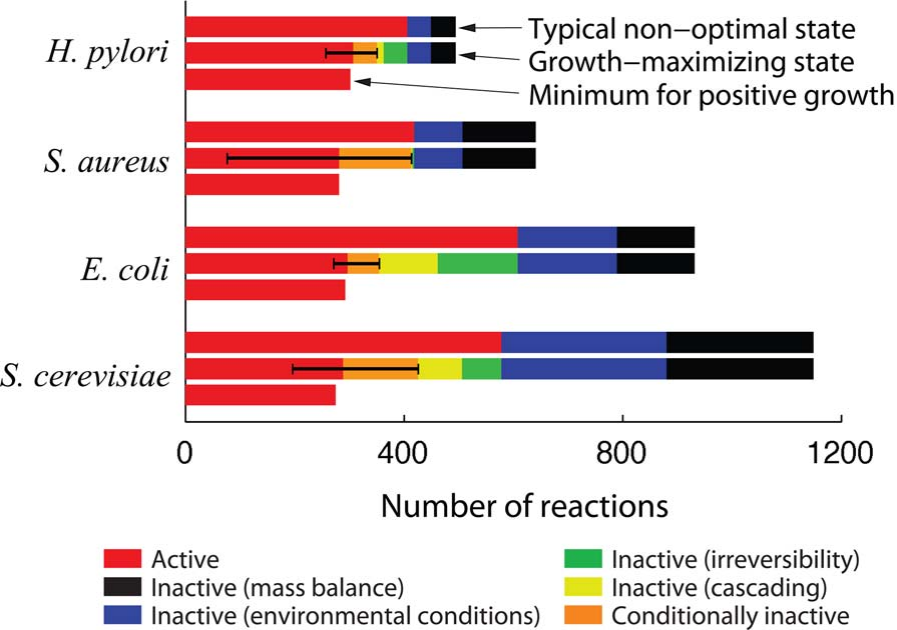
Number of active and inactive reactions in the metabolic networks of *H. pylori*, *S. aureus*, *E. coli*, and *S. cerevisiae*. For each organism, the bars correspond to a typical non-optimal state (top), a growth-maximizing state (middle), and a state with the minimum number of active reactions required for growth (bottom), which was estimated using the algorithm described in Materials and Methods. The error bar represents the upper and lower theoretical bounds, given by Eq. (3), on the number of active reactions in any growth-maximizing state. The breakdown of inactive reactions and their color coding are the same as in Figure 1. All results are obtained using glucose minimal media (Materials and Methods) and are further detailed in Tables 2 and 3.

The results for the typical activity of each organism in glucose minimal media (Materials and Methods) are summarized in the top bars of Figure 2 and in Table 2. The fraction of active reactions ranges from 50%–82%, while 9%–23% are inactive due to mass balance constraints and 9%–26% are inactive due to the environmental conditions. Although the absolute number of active reactions tends to increase with the size of the metabolic network, the fraction of active reactions appears to show the opposite tendency. Figure 1B shows that most of the subsystems of the *E. coli* metabolism are almost completely active, but a few have many inactive reactions. For example, due to the incompleteness of the network many reactions involving cofactors and prosthetic group biosynthesis cannot be used under steady-state conditions in any environment. In addition, many reactions in the alternate carbon metabolism, as well as many transport and extracellular reactions, must be inactive in the absence of the corresponding substrates in the glucose medium.

**Table 2:** Metabolic reactions in typical non-optimal states of the simulated metabolisms.

	*H. pylori*	*S. aureus*	*E. coli*	*S. cerevisiae*
Total number of reactions [*n*]	479	641	931	1149
Inactive reactions:	87	222	322	570
Due to mass balance	44	133	141	268
Due to environmental conditions^a^	43	89	181	302
Active reactions [ 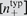 ]	392	419	609	579

a These reactions are inactive due to constraints arising from the availability of substrates in the media defined in Materials and Methods.

### Growth-Maximizing States

We now turn to the maximization of growth rate, which is often hypothesized in flux balance-based approaches and found to be consistent with observation in adaptive evolution experiments [31]–[34]. Performing numerical optimization in glucose minimal media (Materials and Methods), we find that the number of active reactions in such optimal states is reduced by 21%–50% compared to typical non-optimal states, as indicated in the middle bars of Figure 2. Interestingly, the absolute number of active reactions under maximum growth is ∼300 and appears to be fairly independent of the organism and network size for the cases analyzed. We observe that the minimum number of reactions required merely to sustain positive growth [7],[8] is only a few reactions smaller than the number of reactions used in growth-maximizing states (bottom bars, Figure 2). This implies that surprisingly small metabolic adjustment or genetic modification is sufficient for an optimally growing organism to stop growing completely, which reveals a *robust-yet-subtle* tendency in cellular metabolism: while the large number of inactive reactions offers tremendous mutational and environmental robustness[52], the system is very sensitive if limited only to the set of reactions optimally active. The flip side of this prediction is that significant increase in growth can result from very few mutations, as observed recently in adaptive evolution experiments [35].

We now turn to mechanisms underlying the observed reaction silencing, which is spread over a wide range of metabolic subsystems (see Figure 1 for *E. coli*). The phenomenon is caused by growth maximization via reaction irreversibility and cascading of inactivity.

#### Irreversibility

**Figure 3:**
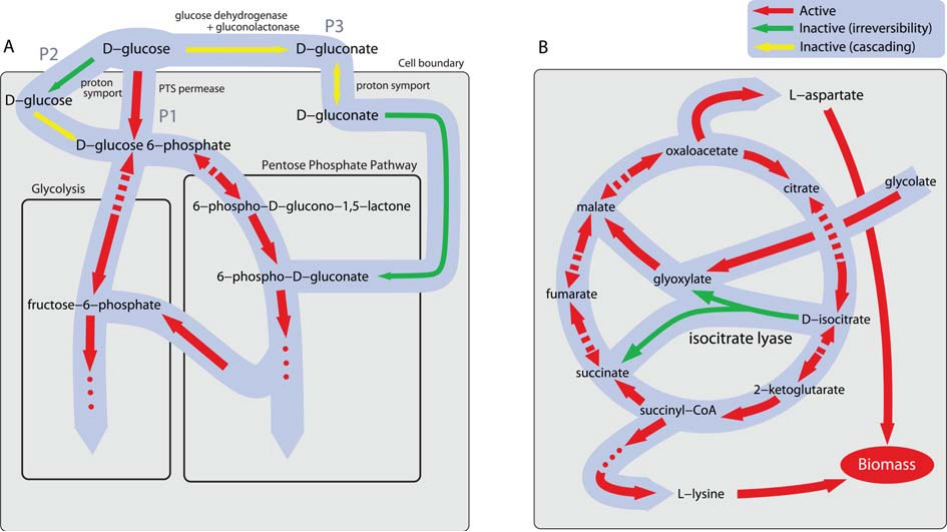
Portions of *E. coli* metabolic network under maximum growth condition. (A) P1, P2, and P3 are alternative pathways for glucose transport and utilization. The most efficient pathway, P1, is active under maximum growth in glucose minimal medium. P2 and P3 are inactive, but if P1 is knocked out, P2 becomes active, and if both P1 and P2 are knocked out, P3 becomes active. In both knockout scenarios, the growth is predicted to be suboptimal. (B) Isocitrate lyase reaction in the pathway bypassing the tricarboxylic acid (TCA) cycle is predicted to be inactive under maximum growth due to its irreversibility. If it were to operate in the opposite direction, it would serve as a *transverse* pathway which redirects metabolic flow to growth-limiting reactions, increasing the maximum biomass production rate slightly. In both panels, single and double arrows are used to indicate irreversible and reversible reactions, respectively, and colors indicate the behavior of the reactions under maximum growth: active (red), inactive due to the irreversibility (green), and inactive due to cascading (yellow).

We identify three different scenarios in which reaction irreversibility causes reaction inactivity under maximum growth. The simplest case is when the reaction is part of a parallel pathway structure. While stoichiometrically equivalent pathways lead to alternate optima [9], “non-equivalent” redundancy can force irreversible reactions in less efficient pathways to be inactive. To illustrate this effect, we show in Figure 3A three alternative pathways (P1, P2, and P3) for glucose transport and utilization in the *E. coli* metabolism. Pathway P1 is active under maximum growth, while P2 and P3 are inactive because they are stoichiometrically less efficient for cellular growth. Indeed, we computationally predict that knocking out P1 would make P2 active, but the growth rate would be reduced by 2.5%. Knocking out both P1 and P2 would make P3 active, but the growth rate would be reduced by more than 10%. Here, the irreversibility of P2 and P3 is essential. For example, if P2 were reversible, the biomass production could be increased (by about 0.05%) by making this pathway active in the opposite direction, which creates a metabolic cycle equivalent to a combination of the pyruvate kinase reaction and the transport of protons out of the cell. The pyruvate kinase itself does not contribute to the increase in biomass production (it is inactive under maximum growth condition), but the cycle would provide a more efficient transport of protons to balance the influx of protons accompanying the ATP synthesis, which in turn would increase biomass production.

A different silencing scenario is identified when no clear parallel pathway structure is recognizable. In this scenario there is a *transverse* pathway that, were it reversible, could be used to increase growth by redirecting metabolic flow from “non-limiting” pathways to those that limit the production of biomass precursors. This includes transverse reactions that establish one-way communication between pathways that lead to different building blocks of the biomass (when one of them is more limiting than the others). In the *E. coli* model, for example, isocitrate lyase in the glyoxylate bypass is predicted to be inactive under maximum growth, as shown in Figure 3B. This prediction is consistent with the observation from growth experiments in glucose media [17]. Again, the irreversibility of the reaction (Note 2) is essential for this argument because, if this constraint is hypothetically relaxed, we predict that the reaction becomes active in the opposite direction, which leads to a slight increase in the maximum growth rate (about 0.005%).

A third scenario for the irreversibility mechanism arises when a transport reaction is irreversible because the corresponding substrate is absent in the medium. For example, since acetate, a possible carbon and energy source, is absent in the given medium, the corresponding transport reaction is irreversible; acetate can only go out of the cell (Note 3). For *E. coli* under maximum growth, we computationally predict that this transport reaction is inactive. This indicates that *E. coli* growing maximally in the given glucose medium wastes no acetate by excretion, which is consistent with experimental observation in glucose-limited culture at low dilution rate [36]. Our predictions in the previous section, in contrast, imply that acetate transport would be active in typical non-optimal states, suggesting that suboptimal growth may induce behavior that mimics acetate overflow metabolism. More generally, we predict that a suboptimal cell will activate *more* transport reactions, and hence excrete *larger* number of metabolites than a growth-optimized cell. This experimentally testable prediction can be further supported by our single-reaction knockout computations considered in a subsequent section (Experimental Evidence) to model suboptimal response to perturbation.

We interpret these inactivation mechanisms involving reaction irreversibility as a consequence of the linear programming property that the set of optimal solutions *M*_opt_ must be part of the boundary of *M* [37]. As such, *M*_opt_ is characterized by a set of *binding* constraints, defined as inequality constraints (e.g., *v_i_*≤*β_i_*) satisfying two conditions: the equality holds (*v_i_* = *β_i_*) for all **v**∈*M*_opt_ and *M*_opt_ is sensitive to changes in the constraints (changes in *β_i_*). In two dimensions, for example, *M*_opt_ would be an edge of *M*, characterized by a single binding constraint, or a corner of *M*, characterized by two binding constraints. In general, at least *d* – *d*_opt_ linearly independent constraints must be binding, where *d* and *d*_opt_ are the dimensions of *M* and *M*_opt_, respectively. Since many metabolic reactions are subject to the irreversibility constraint (*v_i_*≥0), this is expected to lead to many inactive reactions (*v_i_* = 0). Indeed, by excluding the *k* constraints that are not associated with reaction irreversibility (those for the ATP maintenance reaction and exchange fluxes), we obtain an upper bound on the number of active reactions *n*_+_(**v**):
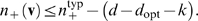
(2)


#### Cascading

The remaining set of reactions that are inactive for all **v**∈*M*_opt_ is due to cascading of inactivity. On one hand, if all the reactions that produce a metabolite are inactive, any reaction that consumes this metabolite must be inactive. On the other hand, if all the reactions that consume a metabolite are inactive, any reaction that produces this metabolite must be inactive to avoid accumulation, as this would violate the steady-state assumption. Therefore, the inactivity caused by the irreversibility mechanism triggers a cascade of inactivity both in the forward and backward directions along the metabolic network. In general, there are many different sets of reactions that, when inactivated, can create the same cascading effect, thus providing flexibility in potential applications of this effect to the design of optimal strains [25]. The cascades in the growth-maximizing states, however, are spontaneous, as opposed to those that would be induced in metabolic knockout applications [25] or in reaction essentiality and robustness studies [38]–[40]. Different but related to the cascades of inactivity are the concepts of enzyme subsets [41], coupled reaction sets [26] and correlated reaction sets [10], which describe groups of reactions that operate together and are thus concurrently inactivated in cascades.

#### Conditional inactivity

While the irreversibility and cascading mechanisms cause the inactivity of many reactions for all **v**∈*M*_opt_, the inactivity of other reactions can depend on the specific growth-maximizing state, whose non-uniqueness in a given environment has been evidenced both theoretically [9],[10],[42] and experimentally [16]. To explore this dependence, we use the duality principle of linear programming problems [37] to identify all the binding constraints generating the set of optimal solutions *M*_opt_ (Text S1, Section 3). This characterization is then used to count the number 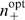 (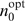) of reactions that are active (inactive) for *all* **v**∈*M*_opt_, leading to rigorous bounds for the number of active reactions *n*_+_(**v**):
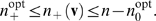
(3)Numerical values of the bounds under maximum growth are indicated by the error bars in Figure 2. Note that the upper bounds are consistently smaller than 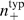 for typical non-optimal states, indicating that reaction silencing necessarily occurs for all growth-maximizing states. For *E. coli*, these results are consistent with a previous study comparing reaction utilization under a range of different growth conditions [10]. They are also consistent with existing results for different *E. coli* metabolic models [12]–[14] based on flux variability analysis [9]. Furthermore, we can prove (Text S1, Section 3) that the distribution of *n*_+_(**v**) within the upper and lower bounds is singular in that the upper bound is attained for almost all optimal states:
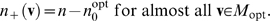
(4)Numerical simulations using standard simplex methods [43] actually result in much fewer active reactions, as shown in Figure 2 (red middle bars), because the algorithm finds solutions on the boundary of *M*_opt_. This behavior is expected, however, under the concurrent optimization of additional metabolic objectives, which generally tend to drive the flux distribution toward the boundary of *M*_opt_.

Figure 2 summarizes the inactivity mechanisms for the four organisms under maximum growth in glucose media (see also Figure 1), showing the inactive reactions caused by the irreversibility (green) and cascading (yellow) mechanisms, as well as those that are conditionally inactive (orange). Observe that in sharp contrast to the number of active reactions, which depends little on the size of the network, the number of inactive reactions (either separated by mechanisms or lumped together) varies widely and shows non-trivial dependence on the organisms.

### Typical Linear Objective Functions

Although we have focused so far on maximizing the biomass production rate, the true nature of the fitness function driving evolution is far from clear [44]–[47]. Organisms under different conditions may optimize different objective functions, such as ATP production or nutrient uptake, or not optimize a simple function at all. In particular, some metabolic behaviors, such as the selection between respiration and fermentation in yeast, cannot be explained by growth maximization [48]. Other behaviors may be systematically different in situations mimicking natural environments [49]. Moreover, various alternative target objectives can be conceived and used in metabolic engineering applications. We emphasize, however, that while specific numbers may differ in each case, all the arguments leading to Eqs. (2)–(4) are general and apply to any objective function that is linear in metabolic fluxes. To obtain further insights, we now study the number of active reactions in organisms optimizing a typical linear objective function by means of random uniform sampling.

We first note the fact (proved in Text S1, Section 4) that with probability one under uniform sampling, the optimal solution set *M*_opt_ consists of a single point, which must be a “corner” of *M*, termed an extreme point in the linear programming literature. In this case, *d*_opt_ = 0, and Eq. (2) becomes
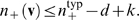
(5)With the additional requirement that the organism show positive growth, we uniformly sample these extreme points, which represent all distinct optimal states for typical linear objective functions.

**Figure 4:**
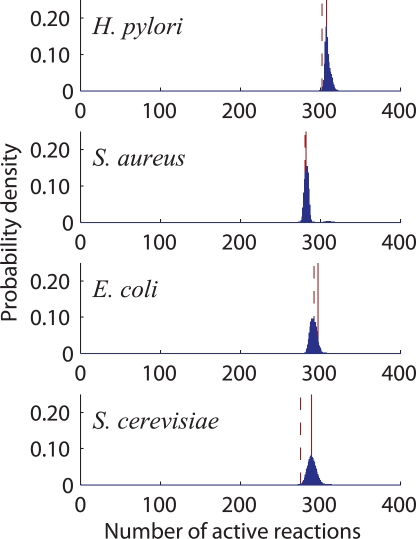
Probability distribution of the number of active reactions in nonzero-growth states that optimize typical objective functions. The red solid lines indicate the corresponding number in the growth-maximizing state of Figure 2 (middle bar, red), and the red dashed lines indicate our estimates of the minimum number of reactions required for the organism to grow (Materials and Methods). [When the nonzero growth requirement is relaxed, a second sharp peak (not shown) arises, corresponding to a drop of ∼250 in the number of active reactions caused by the inactivation of the biomass reaction.]

We find that the number of active reactions in typical optimal states is narrowly distributed around that in growth-maximizing states, as shown in Figure 4. This implies that various results on growth maximization extend to the optimization of typical objective functions. In particular, we see that a typical optimal state is surprisingly close to the onset of cellular growth (estimated and shown as dashed vertical lines in Figure 4). Despite the closeness, however, the organism maintains high *versatility*, which we define as the number of distinct functions that can be optimized under growth conditions. To demonstrate this, consider the *H. pylori* model, which has 392 reactions that *can* be active, among which at least 302 *must* be active to sustain growth (Table 3). While only a few more than 302 active reactions are sufficient to optimize any objective function, the number of combinations for choosing them can be quite large. For example, there are 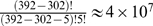 combinations for choosing, say, 5 extra reactions to be active. Moreover, this number increases quickly with the network size: *S. cerevisiae*, for example, has less than 2.5 times more reactions than *H. pylori*, but about 500 times more combinations (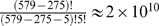).

**Table 3:** Metabolic reactions in maximum growth states of the simulated metabolisms.^a^

	*H. pylori*	*S. aureus*	*E. coli*	*S. cerevisiae*
Active reactions under typical non-optimal states [ 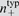 ]	392	419	609	579
Active reactions under maximum growth^b^	308	282	297	289
Lower bound [ 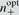 ]	257	77	272	196
Upper bound [ 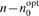 ]	351	414	355	426
Minimum number of active reactions for growth^c^	302	281	292^e^	275
Inactive reactions under maximum growth^b^ [ 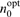 ]:	171	359	634	860
Due to irreversibility	29	3	147	72
Due to cascading	12	2	107	81
Due to mass balance	44	133	141	268
Due to environmental conditions	43	89	181	302
Conditionally inactive^d^	43	132	58	137

a With respect to the minimal media defined in Materials and Methods.

b Based on a single optimal state found using an implementation of the simplex method [43].

c Estimated using the algorithm described in Materials and Methods.

d Predicted to be inactive by the simplex method [43], but can be active in some other growth-maximizing states. Likewise, some of the reactions predicted to be active can be inactive in some other optimal states, but the number of such reactions is expected to be small since the simplex method finds a solution on the boundary of *M*_opt_, which tends to have more inactive reactions than a typical optimal solution.

e The corresponding minimum number of active reactions for maximum growth is 293.

### Experimental Evidence

Our results help explain previous experimental observations. Analyzing the 22 intracellular fluxes determined by Schmidt *et al.* [50] for the central metabolism of *E. coli* in both aerobic and anaerobic conditions, we find that about 45% of the fluxes are smaller than 10% of the glucose uptake rate (Table 4). However, less than 19% of the reversible fluxes and more than 60% of the irreversible fluxes are found to be in this group (Fisher exact test, one-sided *p*<0.008). For the 44 fluxes in the *S. cerevisiae* metabolism experimentally measured by Daran-Lapujade *et al.* [51], less than 8% of the reversible fluxes and more than 42% of the irreversible fluxes are zero (Table 5; Fisher exact test, one-sided *p*<10^−7^). This higher probability for reduced fluxes in irreversible reactions is consistent with our theory and simulation results (Table 6) combined with the assumption that the system operates close to an optimal state. For the *E. coli* data, this assumption is justified by the work of Burgard & Maranas [44], where a framework for inferring metabolic objective functions was used to show that the organisms are mainly (but not completely) driven by the maximization of biomass production. The *S. cerevisiae* data was also found to be consistent with the fluxes computed under the assumption of maximum growth [52].

**Table 4:** Experimentally determined fluxes of intracellular reactions involved in the glycolysis, pentose phosphate pathway, TCA cycle, and amino acid biosynthesis of *E. coli* K12 MG1655 under aerobic and anaerobic conditions [50].

	Aerobic		Anaerobic	
	Reversible	Irreversible	Reversible	Irreversible
Number of fluxes	8	14	8	14
Number of fluxes <10% of glucose uptake rate	1	7	2	10

**Table 5:** Experimentally determined fluxes of intracellular reactions involved in the glycolysis, metabolic steps around pyruvate, TCA cycle, glyoxylate cycle, gluconeogenesis, and pentose phosphate pathway of *S. cerevisiae* strain CEN.PK1137D grown under glucose, maltose, ethanol, and acetate limitation [51].

	Glucose		Maltose		Ethanol		Acetate	
	Rev.	Irr.	Rev.	Irr.	Rev.	Irr.	Rev.	Irr.
Number of fluxes	22	22	22	22	22	22	22	22
Number of zero fluxes	2	8	2	7	1	11	2	11
Percentage of zero fluxes	9.1%	36.4%	9.1%	31.8%	4.5%	50.0%	9.1%	50.0%

**Table 6:** Fraction of inactive reactions in the simulated metabolism of *E. coli* and *S. cerevisiae* under maximum growth condition.^a^

	*E. coli*		*S. cerevisiae*	
	Reversible	Irreversible	Reversible	Irreversible
Number of reactions	245	686	430	719
Number of inactive reactions	139	495	301	559
Percentage of inactive reactions	56.7%	72.2%	70.0%	77.7%

a Same states considered in Table 3.

Additional evidence for our results is derived from the inspection of 18 intracellular fluxes experimentally determined by Emmerling *et al.* [53] for both wild-type *E. coli* and a gene-deficient strain not exposed to adaptive evolution. It has been shown [21] that while the wild-type fluxes can be approximately described by the optimization of biomass production, the genetically perturbed strain operates sub-optimally. We consider the fluxes that are more than 10% (of the glucose uptake rate) larger in the gene-deficient mutant than in the wild-type strain. This group comprises less than 27% of the reversible fluxes but more than 45% of the irreversible fluxes (Table 7; Fisher exact test, one-sided *p*<0.12). This correlation indicates that irreversible fluxes tend to be larger in non-optimal metabolic states, consistently with our predictions.

**Table 7:** Experimentally determined fluxes of reversible and irreversible reactions of wild-type *E. coli* JM101 versus its pyruvate kinase-deficient mutant PB25 [53].

	Reversible	Irreversible
Number of fluxes	30	24
Number of mutant fluxes that are larger^a^ by >10% of glucose uptake rate	8	11

a Relative to the corresponding fluxes in the wild-type strain.

Altogether, our results offer an explanation for the temporary activation of latent pathways observed in adaptive evolution experiments after environmental [16] or genetic perturbations [17]. These initially inactive pathways are transiently activated after a perturbation, but subsequently inactivated again after adaptive evolution. We hypothesize that *transient suboptimal states* are the leading cause of latent pathway activation. Since we predict that a large number of reactions are inactive in optimal states but active in typical non-optimal states, many reactions are expected to show temporary activation *if we assume* that the state following the perturbation is suboptimal and both the pre-perturbation and post-adaptation states are near optimality. To demonstrate this computationally for the *E. coli* model, we consider the idealized scenario where the perturbation to the growth-maximizing wild type is caused by a reaction knockout and the initial response of the metabolic network—before the perturbed strain evolves to a new growth-maximizing state—is well approximated by the method of minimization of metabolic adjustment (MOMA) [21]. MOMA assumes that the perturbed organisms minimize the square-sum deviation of its flux distribution from the wild-type distribution (under the constraints imposed by the perturbation).

**Figure 5:**
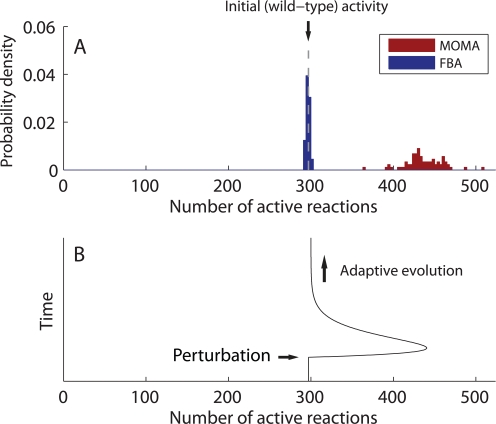
Distribution of the number of active reactions in the *E. coli* metabolic network after a single-reaction knockout. (A) The initial response is predicted by the minimization of metabolic adjustment (MOMA) and the endpoint of adaptive evolution by the maximization of the growth rate (FBA), using the medium defined in Materials and Methods and a commercial optimization software package [79]. We consider all 77 nonlethal single-reaction knockouts that change the flux distribution. (B) Schematic illustration of the network reaction activity during the adaptive evolution after a small perturbation, indicating the temporary activation of many latent pathways.

Figure 5A shows the distribution of the number of active reactions for single-reaction knockouts that alter the flux distribution but allow positive MOMA-predicted growth. While the distribution is spread around 400–500 for the suboptimal states in the initial response, it is sharply peaked around 300 for the optimal states at the endpoint of the evolution, which is consistent with our results on random sampling of the extreme points (Figure 4). We thus predict that the initial number of active reactions for the unperturbed wild-type strain (which is 297, as shown by a dashed vertical line in Figure 5A) typically increases to more than 400 following the perturbation, and then decays back to a number close to 300 after adaptive evolution in the given environment, as illustrated schematically in Figure 5B. A neat implication of our analysis is that the active reactions in the early post-perturbation state includes *much more* than just a superposition of the reactions that are active in the pre- and post-perturbation optimal states, thus explaining the pronounced burst in gene expression changes observed to accompany media changes and gene knockouts [16],[17]. For example, for *E. coli* in glucose minimal medium, temporary activation is predicted for the Entner-Doudoroff pathway after *pgi* knockout and for the glyoxylate bypass after *tpi* knockout, in agreement with recent flux measurements in adaptive evolution experiments [17].

Another potential application of our results is to explain previous experimental evidence that antagonistic pleiotropy is important in adaptive evolution [54], as they indicate that increasing fitness in a single environment requires inactivation of many reactions through regulation and mutation of associated genes, which is likely to cause a decrease of fitness in some other environments [15].

## Discussion

Combining computational and analytical means, we have uncovered the microscopic mechanisms giving rise to the phenomenon of spontaneous reaction silencing in single-cell organisms, in which optimization of a single metabolic objective, whether growth or any other, significantly reduces the number of active reactions to a number that appears to be quite insensitive to the size of the entire network. Two mechanisms have been identified for the large-scale metabolic inactivation: reaction irreversibility and cascade of inactivity. In particular, the reaction irreversibility inactivates a pathway when the objective function could be enhanced by hypothetically reversing the metabolic flow through that pathway. We have demonstrated that such pathways can be found among non-equivalent parallel pathways, transverse pathways connecting structures that lead to the synthesis of different biomass components, and pathways leading to metabolite excretion. Although the irreversibility and cascading mechanisms do not require explicit maximization of efficiency, massive reaction silencing is also expected for organisms optimizing biomass yield or other linear functions (of metabolic fluxes) normalized by uptake rates [18]. Furthermore, while we have focused on minimal media, we expect the effect to be even more pronounced in richer media. On one hand, a richer medium has fewer absent substrates, which increases the number of active reactions in non-optimal states. On the other hand, a richer medium allows the organism to utilize more efficient pathways that would not be available in a minimal medium, possibly further reducing the number of active reactions in optimal states.

Our study carries implications for both natural and engineered processes. In the rational design of microbial enhancement, for example, one seeks genetic modifications that can optimize the production of specific metabolic compounds, which is a special case of the optimization problem we consider here and akin to the problem of identifying optimal reaction activity [24],[25]. The identification of a reduced set of active reactions also provides a new approach for testing the existence of global metabolic objectives and their consistency with hypothesized objective functions [46]. Such an approach is complementary to current approaches based on coefficients of importance [44],[45] or putative objective reactions [47] and is expected to provide novel insights into goal-seeking dynamic concepts such as cybernetic modeling [55]. Our study may also help model compromises between competing goals, such as growth and robustness against environmental or genetic changes [56].

In particular, our results open a new avenue for addressing the origin of mutational robustness [57]–[62]. Single-gene deletion experiments on *E. coli* and *S. cerevisiae* have shown that only a small fraction of their genes are essential for growth under standard laboratory conditions [1],[5],[6]. The number of essential genes can be even smaller given that growth defect caused by a gene deletion may be synthetically rescued by compensatory gene deletions [25], an effect not accounted for in single-gene deletion experiments. Under fixed environmental conditions, large part of this mutational robustness arises from the reactions that are inactive under maximum growth, whose deletion is predicted to have no effect on the growth rate [52]. For example, for *E. coli* in glucose medium, we predict that 638 out of the 931 reactions can be removed simultaneously while retaining the maximum growth rate (Note 4), which is comparable to 686 computed in a minimal genome study in rich media [11]. But what is the origin of all these non-essential genes?

A recent study on *S. cerevisiae* has shown that the single deletion of almost any non-essential gene leads to a growth defect in at least one stress condition [15], providing substantive support for the long-standing hypothesis that mutational robustness is a byproduct of environmental robustness [61] (at least if we assume that the tested conditions are representative of the natural conditions under which the organisms evolved). An alternative explanation would be that in variable environments, which is a natural selective pressure likely to be more important than considered in standard laboratory experiments, the apparently dispensable pathways may play a significant role in suboptimal states induced by environmental changes. This alternative is based on the hypothesis that latent pathways provide intermediate states necessary to facilitate adaptation, therefore conferring competitive advantage *even if the pathways are not active in any single fixed environmental condition*. In light of our results, this hypothesis can be tested experimentally in medium-perturbation assays by measuring the change in growth or other phenotype caused by deleting the predicted latent pathways in advance to a medium change.

We conclude by calling attention to a limitation and strength of our results, which have been obtained using steady-state analysis. Such analysis avoids complications introduced by unknown regulatory and kinetic parameters, but admittedly does not account for constraints that could be introduced by the latter. Nevertheless, we have been able to draw robust conclusions about dynamical behaviors, such as the impact of perturbation and adaptive evolution on reaction activity. Our methodology scales well for genome-wide studies and may prove instrumental for the identification of specific extreme pathways [63],[64] or elementary modes [65],[66] governing the optimization of metabolic objectives. Combined with recent studies on complex networks [67]–[73] and the concept of functional modularity [74], our results are likely to lead to new understanding of the interplay between *network activity* and *biological function*.

### Notes

1. In addition, under steady-state conditions in the media considered in this study, more than 77% of the reversible reactions become constrained to be irreversible, rendering a total of more than 92% of all reactions “effectively” irreversible.
2. This reaction is regarded in the biochemical literature as irreversible under physiological conditions in the cell, and is constrained as such in the modeling literature [14],[32],[75],[76].
3. Similar effective irreversibility is at work for any transport or internal reaction that is a unique producer of one or more chemical compounds in the cell.
4. For single-reaction knockouts, MOMA predicts that 662 out of the 931 deletion mutants grow at more than 99% of the wild-type growth rate. Among these 662 reactions, 95% are predicted to be inactive under maximum growth.

## Materials and Methods

### Strains and Media

All the stoichiometric data for the *in silico* metabolic systems used in our study are available at http://gcrg.ucsd.edu/In_Silico_Organisms. For *H. pylori* 26695 [77], we used a medium with unlimited amount of water and protons, and limited amount of oxygen (5 mmol/g DW-h), L-alanine, D-alanine, L-arginine, L-histidine, L-isoleucine, L-leucine, L-methionine, L-valine, glucose, Iron (II and III), phosphate, sulfate, pimelate, and thiamine (20 mmol/g DW-h). For *S. aureus* N315 [78], we used a medium with limited amount of glucose, L-arginine, cytosine, and nicotinate (100 mmol/g DW-h), and unlimited amount of iron (II), protons, water, oxygen, phospate, sulfate, and thiamin. For *E. coli* K12 MG1655 [75], we used a medium with limited amount of glucose (10 mmol/g DW-h) and oxygen (20 mmol/g DW-h), and unlimited amount of carbon dioxide, iron (II), protons, water, potassium, sodium, ammonia, phospate, and sulfate. For *S. cerevisiae* S288C [76], we used a medium with limited amount of glucose (10 mmol/g DW-h), oxygen (20 mmol/g DW-h), and ammonia (100 mmol/g DW-h), and unlimited amount of water, protons, phosphate, carbon dioxide, potassium, and sulfate. The flux through the ATP maintenance reaction was set to 7.6 mmol/g DW-h for *E. coli* and 1 mmol/g DW-h for *S. aureus* and *S. cerevisiae*.

### Feasible Solution Space

Under steady-state conditions, a cellular metabolic state is represented by a solution of a homogeneous linear equation describing the mass balance condition,
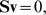
(6)where **S** is the *m*×*N* stoichiometric matrix and 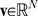 is the vector of metabolic fluxes. The components of **v** = (*v*_1_,…,*v_N_*)*^T^* include the fluxes of *n* internal and transport reactions as well as *n*_ex_ exchange fluxes, which model the transport of metabolites across the system boundary. Constraints of the form *v_i_*≤*β_i_* imposed on the exchange fluxes are used to define the maximum uptake rates of substrates in the medium. Additional constraints of the form *v_i_*≥0 arise for the reactions that are irreversible. Assuming that the cell's operation is mainly limited by the availability of substrates in the medium, we impose no other constraints on the internal reaction fluxes, except for the ATP maintenance flux for *S. aureus*, *E. coli*, and *S. cerevisiae* (see Strains and media section above). The set of all flux vectors **v** satisfying the above constraints defines the *feasible solution space* 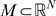, representing the capability of the metabolic network as a system.

### Maximizing Growth and Other Linear Objective Functions

The flux balance analysis (FBA) [18]–[20],[22],[23] used in this study is based on the maximization of a metabolic objective function **c** *^T^* **v** within the feasible solution space *M*, which is formulated as a linear programming problem:
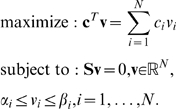
(7)We set *α_i_* = −∞ if *v_i_* is unbounded below and *β_i_* = ∞ if *v_i_* is unbounded above. For a given objective function, we numerically determine an optimal flux distribution for this problem using an implementation of the simplex method [43]. In the particular case of growth maximization, the objective vector **c** is taken to be parallel to the biomass flux, which is modeled as an effective reaction that converts metabolites into biomass.

### Finding Minimum Reaction Set for Nonzero Growth

To find a set of reactions from which none can be removed without forcing zero growth, we start with the set of all reactions and recursively reduce it until no further reduction is possible. At each recursive step, we first compute how much the maximum growth rate would be reduced if each reaction were removed from the set individually. Then, we choose a reaction that causes the least change in the maximum growth rate, and remove it from the set. We repeat this step until the maximum growth rate becomes zero. The set of reactions we have just before we remove the last reaction is a desired minimal reaction set. Note that, since the algorithm is not exhaustive, the number of reactions in this set is an upper bound and approximation for the minimum number of reactions required to sustain positive growth.

## Supporting Information

Text S1 Mathematical Results
(0.06 MB PDF)
