# Supplementary material for: Spontaneous Reaction Silencing in Metabolic Optimization
Source: PLoS Comput Biol. 2008 Dec 5;4(12):e1000236. doi: 10.1371/journal.pcbi.1000236 (PMC2582435; doi:10.1371/journal.pcbi.1000236)
Supplement: Text S1 — Mathematical Results (0.06 MB PDF) [file pcbi.1000236.s001.pdf]

Supporting Information

Text S1: Mathematical Results

1. Number of active reactions in typical steady states

The mass balance constraints $\mathbf{Sv} = \mathbf{0}$ define the linear subspace $\mathrm{Nul}\,\mathbf{S} = \{\mathbf{v} \in \mathbb{R}^N \,|\, \mathbf{Sv} = \mathbf{0}\}$ (the null space of $\mathbf{S}$), which contains the feasible solution space M. However, the set M can possibly be smaller than $\mathrm{Nul}\,\mathbf{S}$ because of the additional constraints arising from the environmental conditions (the availability of substrates in the medium, reaction irreversibility, and cell maintenance requirements). Therefore, M may have smaller dimension than $\mathrm{Nul}\,\mathbf{S}$. If we denote the dimension of M by d, there exists a unique d-dimensional linear submanifold of $\mathbb{R}^N$ that contains M, which we denote by L_M. We can then use the Lebesgue measure naturally defined on L_M to make probabilistic statements, since we can define the probability of a subset $A \subseteq M$ as the Lebesgue measure of A normalized by the Lebesgue measure of M. In particular, we say that $v_i \neq 0$ for *almost all* $\mathbf{v} \in M$ if the set $\{\mathbf{v} \in M \,|\, v_i = 0\}$ has Lebesgue measure zero on L_M. An interpretation of this is that $v_i \neq 0$ with probability one for an organism in a random state under given environmental conditions. Using this notion, we prove the following theorem on the reaction fluxes.

Theorem 1. *If $v_i \neq 0$ for some $\mathbf{v} \in M$, then $v_i \neq 0$ for almost all $\mathbf{v} \in M$.*

Proof. Suppose that $v_i \neq 0$ for some $\mathbf{v} \in M$. The set $L_i := \{\mathbf{v} \in L_M \,|\, v_i = 0\}$ is a linear submanifold of L_M, so we have $\dim L_i \leq \dim L_M$. If $\dim L_i = \dim L_M$, then we have $L_i = L_M \supseteq M$, implying that we have $v_i = 0$ for any $\mathbf{v} \in M$, which violates the assumption. Thus, we must have $\dim L_i < \dim L_M$, implying that L_i has zero Lebesgue measure on L_M. Since $M \subseteq L_M$, we have $M_i := \{\mathbf{v} \in M \,|\, v_i = 0\} \subseteq \{\mathbf{v} \in L_M \,|\, v_i = 0\} = L_i$, and thus M_i also has Lebesgue measure zero. Therefore, we have $v_i \neq 0$ for almost all $\mathbf{v} \in M$. □

Theorem 1 implies that we can group the reactions and exchange fluxes into two categories:

1. *Always inactive*: $v_i = 0$ for all $\mathbf{v} \in M$, and

2. *Almost always active*: $v_i \neq 0$ for almost all $\mathbf{v} \in M$.

Consequently, the number $n_+(\mathbf{v})$ of active reactions satisfies

$$n_+(\mathbf{v}) = n_+^{\text{typ}} := n - n_0^m - n_0^e \quad \text{for almost all } \mathbf{v} \in M, \tag{1}$$

where n_0^m is the number of inactive reactions due to the mass balance constraints (characterized by Theorem 2) and n_0^e is the number of additional reactions in the category 1 above, which are due to the environmental conditions. Combining this result with the finding that optimal states have fewer active reactions (see the main text), it follows that a typical state $\mathbf{v} \in M$ is non-optimal.

2. Inactive reactions due to mass balance constraints

Let us define the stoichiometric coefficient vector of reaction i to be the ith column of the stoichiometric matrix $\mathbf{S}$. We similarly define the stoichiometric coefficient vector of an exchange flux. If the stochiometric vector of reaction i can be written as a linear combination of the stoichiometric vector of reactions/exchange fluxes $i_1, i_2, \ldots, i_k$, we say that i is a linear combination of $i_1, i_2, \ldots, i_k$. We use this linear relationship to completely characterize the set of all reactions that are always inactive due to the mass balance constraints, regardless of any additionally imposed constraints, such as the availability of substrates in the medium, reaction irreversibility, cell maintenance requirements, and optimum growth condition.

Theorem 2. *Reaction i is inactive for all $\mathbf{v}$ satisfying $\mathbf{S}\mathbf{v} = \mathbf{0}$ if and only if it is not a linear combination of the other reactions and exchange fluxes.*

Proof. We denote the stoichiometric coefficient vectors of reactions and exchange fluxes by $\mathbf{s}_1, \ldots, \mathbf{s}_N$. The theorem is equivalent to saying that there exists $\mathbf{v}$ satisfying both $\mathbf{S}\mathbf{v} = \mathbf{0}$ and $v_i \neq 0$ if and only if $\mathbf{s}_i$ is a linear combination of $\mathbf{s}_k$, $k = 1, 2, \ldots, N$, $k \neq i$.

To prove the forward direction in this statement, suppose that $v_i \neq 0$ in a state $\mathbf{v}$ satisfying $\mathbf{S}\mathbf{v} = \mathbf{0}$. By writing out the components of the equation $\mathbf{S}\mathbf{v} = \mathbf{0}$ and rearranging, we get

$$s_{ji} v_i = \sum_{k \neq i} (-v_k) s_{jk}, \quad j = 1, \ldots, m. \tag{2}$$

Since $v_i \neq 0$, we can divide this equation by v_i to see that $\mathbf{s}_i$ is a linear combination of $\mathbf{s}_k$, $k \neq i$ with coefficients $c_k = -v_k/v_i$.

To prove the backward direction, suppose that $\mathbf{s}_i = \sum_{k \neq i} c_k \mathbf{s}_k$. If we choose $\mathbf{v}$ so that $v_k = c_k$ for $k \neq i$ and $v_i = -1$, then for each j, we have

$$(\mathbf{Sv})_j = \sum_k v_k s_{jk} = v_i s_{ji} + \sum_{k \neq i} v_k s_{jk} = -s_{ji} + \sum_{k \neq i} c_k s_{jk} = 0,$$

so $\mathbf{v}$ satisfies $\mathbf{Sv} = \mathbf{0}$. □

3. Number of active reactions in optimal states

The linear programming problem for finding the flux distribution maximizing a linear objective function can be written in the matrix form:

$$\begin{aligned} &\text{maximize:} && \mathbf{c}^T \mathbf{v} \\ &\text{subject to:} && \mathbf{Sv} = \mathbf{0},\ \mathbf{Av} \leq \mathbf{b},\ \mathbf{v} \in \mathbb{R}^N, \end{aligned} \tag{3}$$

where $\mathbf{A}$ and $\mathbf{b}$ are defined as follows. If the ith constraint is $v_j \leq \beta_j$, the ith row of $\mathbf{A}$ consists of all zeros except for the jth entry that is 1, and $b_i = \beta_j$. If the ith constraint is $\alpha_j \leq v_j$, the ith row of $\mathbf{A}$ consists of all zeros except for the jth entry that is -1, and $b_i = -\alpha_j$. A constraint of the type $\alpha_j \leq v_j \leq \beta_j$ is broken into two separate constraints and represented in $\mathbf{A}$ and $\mathbf{b}$ as above. The inequality between vectors is interpreted as inequalities between the corresponding components, so if the rows of $\mathbf{A}$ are denoted by $\mathbf{a}_1^T, \mathbf{a}_2^T, \ldots, \mathbf{a}_K^T$ (where $\mathbf{a}_i^T$ denotes the transpose of $\mathbf{a}_i$), $\mathbf{Av} \leq \mathbf{b}$ represents the set of K constraints $\mathbf{a}_i^T \mathbf{v} \leq b_i$, $i = 1, \ldots, K$. By defining the feasible solution space

$$M := \{\mathbf{v} \in \mathbb{R}^N \,|\, \mathbf{Sv} = \mathbf{0},\ \mathbf{Av} \leq \mathbf{b}\}, \tag{4}$$

the problem can be compactly expressed as maximizing $\mathbf{c}^T \mathbf{v}$ in M.

The duality principle (Best & Ritter, 1985) expresses that any linear programming problem (primal problem) is associated with a complementary linear programming problem (dual problem), and the solutions of the two problems are intimately related. The dual problem associated

with problem (3) is

$$\begin{aligned} \text{minimize:} \quad & \mathbf{b}^T \mathbf{u}_1 \\ \text{subject to:} \quad & \mathbf{A}^T \mathbf{u}_1 + \mathbf{S}^T \mathbf{u}_2 = \mathbf{c},\ \mathbf{u}_1 \geq \mathbf{0}, \\ & \mathbf{u}_1 \in \mathbb{R}^K,\ \mathbf{u}_2 \in \mathbb{R}^m, \end{aligned} \tag{5}$$

where $\{\mathbf{u}_1, \mathbf{u}_2\}$ is the dual variable. A consequence of the Strong Duality Theorem (Best & Ritter, 1985) is that the primal and dual solutions are related via a well-known optimality condition: $\mathbf{v}$ is optimal for problem (3) if and only if there exists $\{\mathbf{u}_1, \mathbf{u}_2\}$ such that

$$\mathbf{S}\mathbf{v} = \mathbf{0},\ \mathbf{A}\mathbf{v} \leq \mathbf{b}, \tag{6}$$

$$\mathbf{A}^T \mathbf{u}_1 + \mathbf{S}^T \mathbf{u}_2 = \mathbf{c},\ \mathbf{u}_1 \geq \mathbf{0}, \tag{7}$$

$$\mathbf{u}_1^T (\mathbf{A}\mathbf{v} - \mathbf{b}) = 0. \tag{8}$$

Note that each component of $\mathbf{u}_1$ can be positive or zero, and we can use this information to find a set of reactions that are forced to be inactive under optimization, as follows. For any given optimal solution $\mathbf{v}_0$, Eq. (8) is equivalent to $u_{1i}(\mathbf{a}_i^T \mathbf{v}_0 - b_i) = 0$, $i = 1, \ldots, K$, where u_{1i} is the ith component of $\mathbf{u}_1$. Thus, if $u_{i1} > 0$ for a given i, we have $\mathbf{a}_i^T \mathbf{v}_0 = b_i$, and we say that the constraint $\mathbf{a}_i^T \mathbf{v} \leq b_i$ is *binding* at $\mathbf{v}_0$. In particular, if an irreversible reaction ($v_i \geq 0$) is associated with a positive dual variable ($u_{1i} > 0$), then the irreversibility constraint is binding, and the reaction is inactive ($v_i = 0$) at $\mathbf{v}_0$. In fact, we can say much more: we prove the following theorem stating that such a reaction is actually *required to be inactive for all possible optimal solutions* for a given objective function $\mathbf{c}^T \mathbf{v}$.

Theorem 3. *Suppose* $\{\mathbf{u}_1, \mathbf{u}_2\}$ *is a dual solution corresponding to an optimal solution of problem* (3). *Then, the set* M_{opt} *of all optimal solutions of* (3) *can be written as*

$$M_{opt} = \{\mathbf{v} \in M \mid \mathbf{a}_i^T \mathbf{v} = b_i \text{ for all } i \text{ for which } u_{1i} > 0\}, \tag{9}$$

and hence every reaction associated with a positive dual component is binding for all optimal solutions in M_{opt}.

Sketch of proof. Let $\mathbf{v}_0$ be the optimal solution associated with $\{\mathbf{u}_1, \mathbf{u}_2\}$ and let Q denote the right hand side of (9). Any $\mathbf{v} \in Q$ is an optimal solution of (3), since straightforward verification shows that it satisfies (6-8) with the same dual solution $\{\mathbf{u}_1, \mathbf{u}_2\}$. Thus, we have $Q \subseteq M_{\text{opt}}$.

Conversely, suppose that $\mathbf{v}$ is an optimal solution of (3). Then, $\mathbf{v}$ can be shown to belong to H, which we define to be the hyperplane that is orthogonal to $\mathbf{c}$ and contains $\mathbf{v}_0$, i.e.,

$$H := \{\mathbf{v} \in \mathbb{R}^N \mid \mathbf{c}^T(\mathbf{v} - \mathbf{v}_0) = 0\}. \tag{10}$$

This, together with the fact that $\mathbf{v}$ satisfies $\mathbf{Sv} = \mathbf{0}$ and $\mathbf{Av} \leq \mathbf{b}$, from (6), can be used to show that $\mathbf{v} \in Q$. Therefore, any optimal solution must belong to Q. Putting both directions together, we have $Q = M_{\text{opt}}$. □

Thus, once we solve Eq. (3) numerically and obtain a *single* pair of primal and dual solutions ($\mathbf{v}_0$ and $\{\mathbf{u}_1, \mathbf{u}_2\}$), we can use the characterization of M_{opt} given in Eq. (9) to identify all reactions that are required to be inactive (or active) for any optimal solutions. To do this we solve the following auxiliary linear optimization problems for each $i = 1, \ldots, N$:

$$\begin{aligned} &\text{maximize/minimize:} && v_i \\ &\text{subject to:} && \mathbf{Sv} = \mathbf{0},\ \mathbf{Av} \leq \mathbf{b},\ \mathbf{a}_i^T\mathbf{v} = b_i \text{ for all } i \text{ for which } u_{1i} > 0. \end{aligned} \tag{11}$$

If the maximum and minimum of v_i are both zero, then the corresponding reaction is required to be inactive for all $\mathbf{v} \in M_{\text{opt}}$. If the minimum is positive or maximum is negative, then the reaction is required to be active. Otherwise, the reaction may be active or inactive, depending on the choice of an optimal solution. Thus, we obtain the numbers n_+^{opt} and n_0^{opt} of reactions that are required to be active and inactive, respectively, for all $\mathbf{v} \in M_{\text{opt}}$. The number of active reactions for any $\mathbf{v} \in M_{\text{opt}}$ is then bounded as

$$n_+^{\text{opt}} \leq n_+(\mathbf{v}) \leq n - n_0^{\text{opt}}. \tag{12}$$

The distribution of $n_+(\mathbf{v})$ within the bounds is singular: the upper bound in Eq. (12) is attained for almost all $\mathbf{v} \in M_{\text{opt}}$. To see this, we apply Theorem 1 with M replaced by M_{opt}. This is justified since we can obtain M_{opt} from M by simply imposing additional equality constraints. Therefore, if we set aside the n_0^{opt} reactions that are required to be inactive (including n_0^m and n_0^e reactions that are inactive for all $\mathbf{v} \in M$), all the other reactions are active for almost all $\mathbf{v} \in M_{\text{opt}}$. Consequently,

$$n_+(\mathbf{v}) = n - n_0^{\text{opt}} \quad \text{for almost all } \mathbf{v} \in M_{\text{opt}}. \tag{13}$$

We can also use Theorem 3 to further classify those inactive reactions caused by the optimization as due to two specific mechanisms:

1. **Irreversibility.** The irreversibility constraint ($v_i \geq 0$) on a reaction can be binding ($v_i = 0$), which directly forces the reaction to be inactive for all optimal solutions. Such inactive reactions are identified by checking the positivity of dual components (u_{1i}).

2. **Cascading.** All other reactions that are required to be inactive for all $\mathbf{v} \in M_{\text{opt}}$ are due to a cascade of inactivity triggered by the first mechanism, which propagates over the metabolic network via the stoichiometric and mass balance constraints.

In general, a given solution of problem (3) can be associated with multiple dual solutions. The set and the number of positive components in $\mathbf{u}_1$ can depend on the choice of a dual solution, and therefore the categorization according to mechanism is generally not unique. As an example, consider a metabolic network containing a chain of two simple irreversible reactions, $A \xrightarrow{v_1} B \xrightarrow{v_2} C$. Since the two reactions are fully coupled via the mass balance constraint ($v_1 = v_2$ whenever $\mathbf{S}\mathbf{v} = \mathbf{0}$), we can show that different combinations of dual components are possible for a given optimal solution: (i) $u_{11} > 0, u_{12} = 0$; (ii) $u_{11} = 0, u_{12} > 0$; or (iii) $u_{11} > 0, u_{12} > 0$. In each case, the set of reactions in the irreversibility category is different, and the number of such reactions are different in case (iii). This comes from the fact that the same result ($v_1 = v_2 = 0$) follows from forcing $v_1 = 0$ only, $v_2 = 0$ only, or both. Thus, we can interpret the non-uniqueness of the categorization as the fact that different sets of triggering inactive reactions can create the same cascading effect on the reaction activity.

4. Typical linear objective functions

Since the feasible solution space M is convex, its "corner" can be mathematically formulated as an *extreme point*, defined as a point $\mathbf{v} \in M$ that cannot be written as $\mathbf{v} = a\mathbf{x} + b\mathbf{y}$ with $a + b = 1$, $0 < a < 1$ and $\mathbf{x}, \mathbf{y} \in M$ such that $\mathbf{x} \neq \mathbf{y}$. Intuition from the two-dimensional case (Fig. S1) suggests that for a typical choice of the objective vector $\mathbf{c}$ such that Eq. (3) has a solution, the solution is unique and located at an extreme point of M. We prove here that

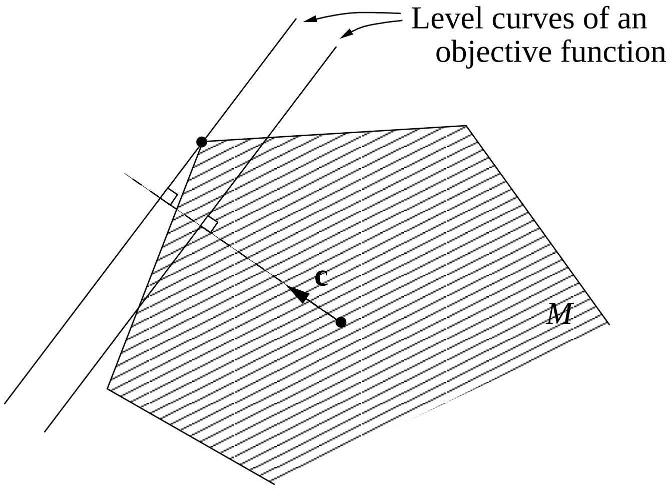

Figure S1: Optimum is typically achieved at a single extreme point. The only exception is when the objective vector $\mathbf{c}$ is in the direction perpendicular to an edge, in which case all points on the edge are optimal.

this is indeed true in general, as long as the objective function is bounded on M, and hence an optimal solution exists.

Theorem 4. *Suppose that the set of objective vectors $B = \{\mathbf{c} \in \mathbb{R}^N \mid \mathbf{c}^T\mathbf{v}$ is bounded on $M\}$ has positive Lebesgue measure. Then, for almost all $\mathbf{c}$ in B, there is a unique solution of Eq.* (3)*, and it is located at an extreme point of M.*

Proof. For a given $\mathbf{c} \in B$, the function $\mathbf{c}^T\mathbf{v}$ is bounded on M, so the solution set $M_{\text{opt}} = M_{\text{opt}}(\mathbf{c})$ of Eq. (3) consists of either a single point or multiple points. Suppose M_{opt} consists of a single point $\mathbf{v}$ and it is not an extreme point. By definition, it can be written as $\mathbf{v} = a\mathbf{x} + b\mathbf{y}$ with $a + b = 1$, $0 < a < 1$ and $\mathbf{x}, \mathbf{y} \in M$ such that $\mathbf{x} \neq \mathbf{y}$. Since $\mathbf{v}$ is the only solution of Eq. (3), $\mathbf{x}$ and $\mathbf{y}$ must be suboptimal, and hence we have $\mathbf{c}^T\mathbf{x} < \mathbf{c}^T\mathbf{v}$ and $\mathbf{c}^T\mathbf{y} < \mathbf{c}^T\mathbf{v}$. Then,

$$\begin{aligned}
\mathbf{c}^T\mathbf{y} &= \mathbf{c}^T(\mathbf{v} - a\mathbf{x})/b \\
&= (\mathbf{c}^T\mathbf{v} - a\mathbf{c}^T\mathbf{x})/b \\
&> (\mathbf{c}^T\mathbf{v} - a\mathbf{c}^T\mathbf{v})/b \\
&= \frac{1-a}{b}\,\mathbf{c}^T\mathbf{v} \\
&= \mathbf{c}^T\mathbf{v},
\end{aligned}$$

and we have a contradiction with the fact that $\mathbf{v}$ is an optimum. Therefore, if M_{opt} consists of a single point, it must be an extreme point of M.

We are left to show that the set of $\mathbf{c} \in B$ for which $M_{\text{opt}}(\mathbf{c})$ consists of multiple points has Lebesgue measure zero. By Theorem 3, for a given $\mathbf{c}$, there exists a set of indices $I \subseteq \{1, \ldots, K\}$ such that $M_{\text{opt}}(\mathbf{c}) = Q_I := \{\mathbf{v} \in M \mid \mathbf{a}_i^T \mathbf{v} = b_i \text{ for all } i \in I\}$, so

$$\{\mathbf{c} \in \mathbb{R}^N \mid M_{\text{opt}}(\mathbf{c}) \text{ contains multiple points}\} \subseteq \bigcup_I \{\mathbf{c} \in \mathbb{R}^N \mid Q_I = M_{\text{opt}}(\mathbf{c})\}, \qquad (14)$$

where the union is taken over all $I \subseteq \{1, \ldots, K\}$ for which Q_I contains multiple points. If $\mathbf{c}$ is in one of the sets in the union in Eq. (14), the set Q_I, being the set of all optimal solutions, is orthogonal to $\mathbf{c}$. Hence, $\mathbf{c}$ is in $Q_I^\perp$, the orthogonal complement of Q_I defined as the set of all vectors orthogonal to Q_I. Therefore,

$$\{\mathbf{c} \in \mathbb{R}^N \mid M_{\text{opt}}(\mathbf{c}) \text{ contains multiple points}\} \subseteq \bigcup_I Q_I^\perp, \qquad (15)$$

Because Q_I is convex, it contains multiple points if and only if its dimension is at least one, implying that each $Q_I^\perp$ in the union in Eq. (15) has dimension at most $N - 1$, and hence has zero Lebesgue measure in $\mathbb{R}^N$. Since there are only a finite number of possible choices for $I \subseteq \{1, \ldots, K\}$, the right hand side of Eq. (15) is a finite union of sets of Lebesgue measure zero. Therefore, the left hand side also has Lebesgue measure zero. □

Reference

Best MJ, Ritter K (1985) *Linear Programming: Active Set Analysis and Computer Programs.* Prentice-Hall, Engelwood Cliffs, New Jersey, USA
